# Speciation and Extinction Drive the Appearance of Directional Range Size Evolution in Phylogenies and the Fossil Record

**DOI:** 10.1371/journal.pbio.1001260

**Published:** 2012-02-21

**Authors:** Alex L. Pigot, Ian P. F. Owens, C. David L. Orme

**Affiliations:** 1 Edward Grey Institute, Department of Zoology, University of Oxford, Oxford, United Kingdom; 2 Division of Biology, Department of Life Sciences, Imperial College London, Ascot, United Kingdom; 3 Grantham Institute for Climate Change, Imperial College London, London, United Kingdom; 4 Natural History Museum, London, United Kingdom; University of California Berkeley, United States of America

## Abstract

While the geographic range of a species is a fundamental unit of macroecology and a leading predictor of extinction risk, the evolutionary dynamics of species' ranges remain poorly understood. Based on statistical associations between range size and species age, many studies have claimed support for general models of range evolution in which the area occupied by a species varies predictably over the course of its life. Such claims have been made using both paleontological data and molecular estimates of the age of extant species. However, using a stochastic model, we show that the appearance of trends in range size with species' age can arise even when range sizes have evolved at random through time. This occurs because the samples of species used in existing studies are likely to be biased with respect to range size: for example, only those species that happened to have large or expanding ranges are likely to survive to the present, while extinct species will tend to be those whose ranges, by chance, declined through time. We compared the relationship between the age and range size of species arising under our stochastic model to those observed across 1,269 species of extant birds and mammals and 140 species of extinct Cenozoic marine mollusks. We find that the stochastic model is able to generate the full spectrum of empirical age–area relationships, implying that such trends cannot be simply interpreted as evidence for models of directional range size evolution. Our results therefore challenge the theory that species undergo predictable phases of geographic expansion and contraction through time.

## Abstract 2

The appearance of directional trends in the evolution of species range sizes can arise from stochastic models and need not imply the existence of underlying trends.

## Author Summary

The changing distribution of species over the surface of the Earth, likened by Darwin to “a grand game of chess with the World for a board”, is a central theme in the evolution of life. Studies of the fossil record and molecular estimates of species ages appear to show that species follow a predictable trend of expansion and contraction of their geographic range over evolutionary time. However, using computer simulations we show that the observed correlation between the age of a species and the extent of its range is expected even if changes in range area have occurred randomly through time. Our results cast doubt on the evidence for directional trends in the long-term evolution of species ranges and suggest that the general rules governing this grand game may need to be re-evaluated.

## Introduction

The geographic area occupied by a species is known to vary through time [1],[2], but whether these dynamics follow any regular trends or are instead largely idiosyncratic remains controversial [3]–[6]. A number of recent studies, however, have revealed a remarkable pattern whereby the relative range size of a species does appear to vary predictably with its evolutionary age [7]–[15]. The results of these studies are intriguing because they suggest that species have a geographical ontogeny, akin to the life cycle of an individual organism [14]. Similar ideas have been proposed many times before [1],[2],[4],[16]–[19]. For example, in Willis's [1] theory of “age and area”, the geographic range of a species continues to expand over the course of its life, while in the “taxon cycle”, newly formed species rapidly expand their distributions before undergoing a protracted decline that ends either in extinction or the initiation of a new wave of the cycle [4],[17].

In the fossil record, where the trajectories of individual lineages can be traced through time, the predominant trend appears to be for newly formed species to gradually expand their ranges, only to later undergo a gradual decline to extinction. This pattern of “rise and fall” has been reported from a taxonomically broad set of groups including mammals [10],[20], plankton [8],[9], and marine mollusks [7]. Molecular phylogenies have also been used to infer the mode of range evolution within lineages by comparing the range sizes of extant species of varying age, where age is estimated as the time since divergence from the closest extant relative [11] (for an alternative method of inferring range evolution see [21],[22]). Such “age–area” correlations have been tested in a wide variety of groups including birds [11],[14], mammals [12], plants [13], frogs [15], aquatic beetles [23], and reef fish [24]. A pattern emerging from these studies is that no single model of range evolution appears to apply across groups [3]: while in some cases range size is independent of species age, in others there is evidence for increasing, decreasing, or cyclical range size trends.

The assumption underlying all these previous studies is that, in the absence of any tendency for ranges to either expand or contract, range size and species age should be independent [7],[8],[11]. Evidence for directional trends in range evolution is therefore provided when the slope of the relationship between age and area departs significantly from zero. However this assumption may be violated under a model of stochastic range evolution, speciation, and extinction. This is because, in phylogenies containing only extant species, range sizes may increase with age simply because species with small or declining ranges are less likely to have survived to the present [25],[26]. Further biases may also be expected because of the interactions between geographic range size and the process of speciation [22],[27]. Even in the fossil record, the appearance of regular trends in range size could arise if studies restrict their analysis to those species that go extinct before the present [7]–[9] or if species with small geographic ranges are less likely to be sampled [28]–[30].

To examine the extent to which these processes could account for the patterns observed between range size and species age, we developed a stochastic model of range evolution that incorporates the effects of speciation and extinction on geographic range size [27],[31]–[33]. We then compared the age–area correlations arising from this model to those observed across the tips of 39 avian and mammalian molecular phylogenies containing 1,269 species from across 64 genera and 17 orders. These groups provide an ideal case study because there are abundant data on their phylogenetic relationships and geographic distributions [34]–[36], and as a result they have also featured prominently in previous studies reporting trends between range size and evolutionary age (e.g., [12],[14],[37]). Finally, we tested the extent to which the null model can account for the patterns observed in the fossil record using the occupancy trajectories of 140 species of extinct marine mollusks [7]. This dataset was previously presented as evidence that species exhibit regular patterns of range size evolution [7], but a departure from a stochastic model has not yet been tested. We demonstrate that under a stochastic model, range size and species age are not expected to be independent and that correlations between age and area observed in phylogenies and the fossil record therefore need not imply a deterministic trend in range size evolution within lineages.

## Results

### Age–Area Relationships Expected under a Stochastic Model of Range Size Evolution

We modelled a process of range evolution and species diversification in which range sizes within lineages evolved according to a random walk through time (see Materials and Methods for details). Within the simulation, we considered two models of speciation rate (either independent or positively correlated with range size) and varied both the rate of change in range size and the asymmetry of range division amongst the daughter lineages during speciation (see Materials and Methods for details). Extinction occurs when the range size of a species walks to or below zero. For each combination of speciation model, rate of range evolution, and range inheritance asymmetry, we ran 500–5,000 replicates of clade diversification and range evolution.

**Figure 1:**
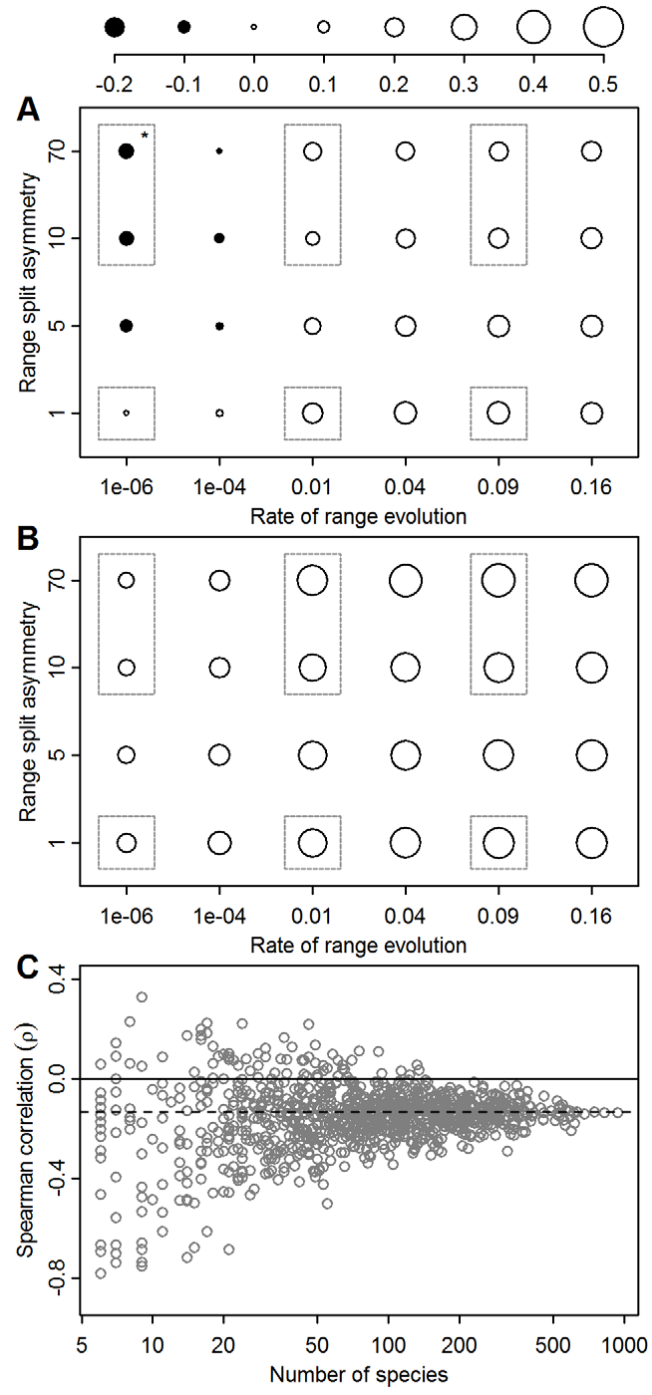
The relationship between the range size and the age of extant species expected under the stochastic model. Variation in Spearman's correlation (ρ) between species' age and geographic range area under different combinations of asymmetry in range size inheritance and rate of change in range size where probability of speciation (ν) increases with range size (A) or is constant (B). Filled circles indicate negative correlations, while unfilled circles indicate positive correlations. Correlations are across all simulated clades for a particular parameter combination. Using a subsample of clades within an example combination (marked with an asterisk) shows that the observed correlation is strongly dependent on clade size (C). Grey boxes highlight the area of parameter space presented in Figure 2.

Pooling the results from across replicate clades shows that range size may show strong correlations with species age even under a random model of range size evolution (Figure 1). Range size is positively correlated with evolutionary age across much of the parameter space, and it is evident that high rates of range evolution give rise to stronger positive correlations (Figure 1, unfilled circles), resulting from increased extinction of small-ranged species. In rare circumstances, our stochastic model predicts negative age–area correlations (Figure 1, filled circles). This requires slow rates of range evolution, an increase in the probability of speciation with range size, and high asymmetry in range splitting (Figure 1A). Under this scenario, which resembles a peripatric speciation model [38], small-ranged species are less likely to speciate than large-ranged species but are also unlikely to go extinct. They will therefore tend to occur on the end of longer terminal branches than large-ranged species [27],[39]. In contrast, when speciation rate is independent of range size (Figure 1B), positive age–area correlations are expected even when extinction is negligible. This occurs because range sizes decrease at speciation, and so species with the largest ranges will tend to be those that have not recently undergone speciation.

Although these correlations reveal the overall expected relationship under different scenarios, individual simulations exhibit considerable variation in the correlation between age and area, and pooling smaller sets of simulations shows the extent of this variation (Figure 1C). Therefore, we also calculated the relative frequencies with which individual simulations fall into five broad categories of age–area relationships. To do this, we fitted range size as a quadratic function of species age for each clade in our simulated dataset, using F-tests of the significance of terms to simplify to a linear regression or a null model where appropriate. We aggregated the results of these models into five broad classes: no relationship, increasing relationship, decreasing relationship, intermediate peaks, and intermediate troughs (see Materials and Methods for details) (Figure S1). Our results were qualitatively similar when using more detailed curve classifications (Figures S1 and S4; Table S1).

### Observed Age–Area Correlations among Extant Species

We assessed the age–area relationships of individual genera of birds and mammals using the same curve classification procedure as for our simulated clades. Observed clades exhibit a variety of relationships, including positive linear and intermediate troughs, but in only a minority of cases are these age–area relationships significant (9.4%; Tables 1, S1, and S2). In accordance with the effects of sample size on statistical power revealed by our simulations (Figure 1C), grouping species into orders (median richness = 29 species) rather than genera (median richness = 12 species) increases the proportion of clades exhibiting significant age–area correlations to 23.5% (Table 1).

**Table 1:** Age–area relationships across clades of birds and mammals.

Age–Area Class	Genera	Orders
–	90.6 (93.3, 88.2)	76.5 (75, 77.8)
/	4.7 (6.7, 2.94)	11.8 (0, 22.2)
\	0 (0, 0)	0 (0, 0)
∩	0 (0, 0)	0 (0, 0)
∩	4.7 (0, 8.82)	11.8 (25, 0)
*N*	64 (30, 34)	17 (8, 9)
*S*	12 (9.5, 12)	29 (59, 24)

The percentage of total taxa falling into each relationship class is given, along with the percentages of mammal and bird taxa in parentheses. Results are presented for individual genera and for genera combined into orders, along with the number of clades (*N*) and their median species richness (*S*). See Figure S1 for classification of age–area relationships and Table S2 for results for individual clades.

### Observed versus Simulated Age–Area Correlations in Extant Species

**Figure 2:**
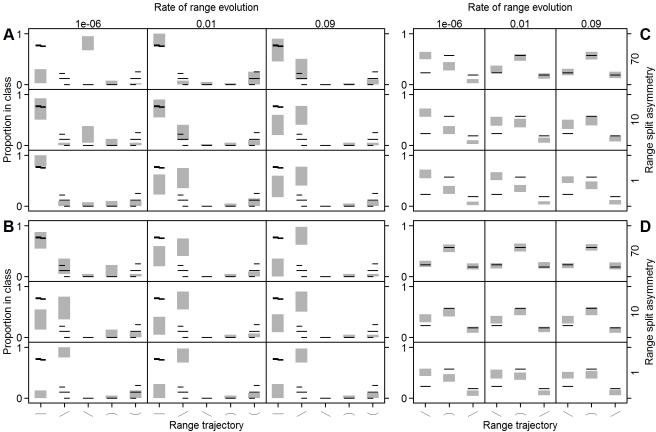
The relative proportions of observed and expected age–area trajectories across reconstructed phylogenies and extinct species. The grey bars show the 95% confidence limits of the expected relative proportion of different age–area relationship classes under different combinations of asymmetry and range size inheritance for extant vertebrates (A and B) and extinct molluscs (C and D). The probability of speciation (ν) increases with range size (A and C) or is constant (B and D). Observed proportions of each class are shown as black bars for vertebrate orders (Table 1) and mollusk species (Table S3); shorter bars in (A) and (B) show the proportions for bird (left) and mammal (right) orders separately. The nine panels in each block correspond to the highlighted areas of Figure 1.

Different combinations of parameter values in the null model give rise to differences in the relationship expected between range size and evolutionary age (Figures 1, 2A, and 2B). One explanation for the variety of age–area correlations observed in the empirical data is therefore that rates of range evolution or the geographic mode of speciation have differed amongst clades. However, when clade sizes are relatively small, as is typical of empirical datasets, we expect to see substantial variation in age–area correlations simply because of chance. To explore this effect, for each point in parameter space, we compared the proportion of observed and simulated clades exhibiting a particular age–area correlation. Because of the strong effect of clade richness on the patterns (Figure 1C), we randomly aggregated our simulated clades until average species richness was similar to that of our empirical dataset. The results for a representative sample of parameter space show that the full spectrum of observed age–area correlations can often arise because of stochastic sampling of the overall null expectation (Figure 2A and 2B). However, the precise frequency of the different age–area curves across avian and mammalian clades cannot generally be explained by any single combination of parameters (Figure 2A and 2B). Overall the results suggest that while substantial variation in age–area correlations observed across clades may be due to chance, differences in the rates of range evolution or modes of geographic speciation across clades may also be required.

### Range Size Trajectories of Extinct Species in the Fossil Record and the Stochastic Model

To investigate whether the stochastic model can account for the patterns observed in the fossil record, we extracted the extinct lineages from our simulations and assigned each one of these to a range size trajectory. To ensure that the definition of species age in our simulations was consistent with the empirical data, we measured absolute species ages under a model of ancestral persistence: upon each speciation event the lineage with the larger range size retained the ancestral species name, with the smaller ranged lineage designated as a new species. Species ages thus represent the time between the first and last occurrence of a species and are not affected by the speciation or extinction of other lineages [40].

The empirical dataset consists of the occupancy trajectories of extinct marine mollusks provided by Foote et al. [7]. Because many (44%) of the species occurred in only three statigraphic stages we did not attempt to fit curves to these and instead assigned each species to one of three possible range trajectories, depending on whether its peak mean range size was reached in the first, second, or third tercile of its life (see Materials and Methods for details). If range size is independent of evolutionary age, then a similar proportion of species should reach their maximum extent across each of the three sampling intervals. We used exactly the same procedure for assigning range size trajectories in our simulated dataset.

We found that approximately 57% of mollusk species reached their peak range size in the middle of their lives, with the number of peaks in the first and final third relatively evenly split (Figure 2C and 2D; Table S3). Our analysis therefore supports the pattern of “rise and fall” previously reported for this group (multinomial model: *p*<0.017). However, our stochastic model shows that when geographic ranges have evolved randomly through time, range size is not expected to be independent of species age (Figure 2C and 2D). Instead, most species are expected to reach their maximum geographic extent in either the first or second interval of their lives, and typically have smaller ranges at the end of their durations (Figure 2C and 2D). This occurs because extinct species must have undergone a net decline in range size through time, and, when ranges evolve according to a random walk, extinction is likely to be preceded by rarity unless rates of range evolution are extremely high.

Whether species undergo a continuous decline or exhibit an intermediate peak depends on the probability of extinction amongst newly formed species (Figure 2C and 2D). When young species have a low probability of extinction, either because they inherit a large range or because ranges are relatively stable, then the predominant pattern is for range sizes to simply decline through time (Figure 2C and 2D). In contrast, when species inherit a small geographic range or when rates of range evolution are high, then only those species that initially expand their distributions are likely to persist for a sufficient length of time to be included in the analysis (i.e., more than two time steps; see Materials and Methods for details) (Figure 2C and 2D). Under these conditions the relative frequency of the different range size trajectories observed across marine mollusks is consistent with that expected under a stochastic model (Figure 2C and 2D).

## Discussion

**Figure 3:**
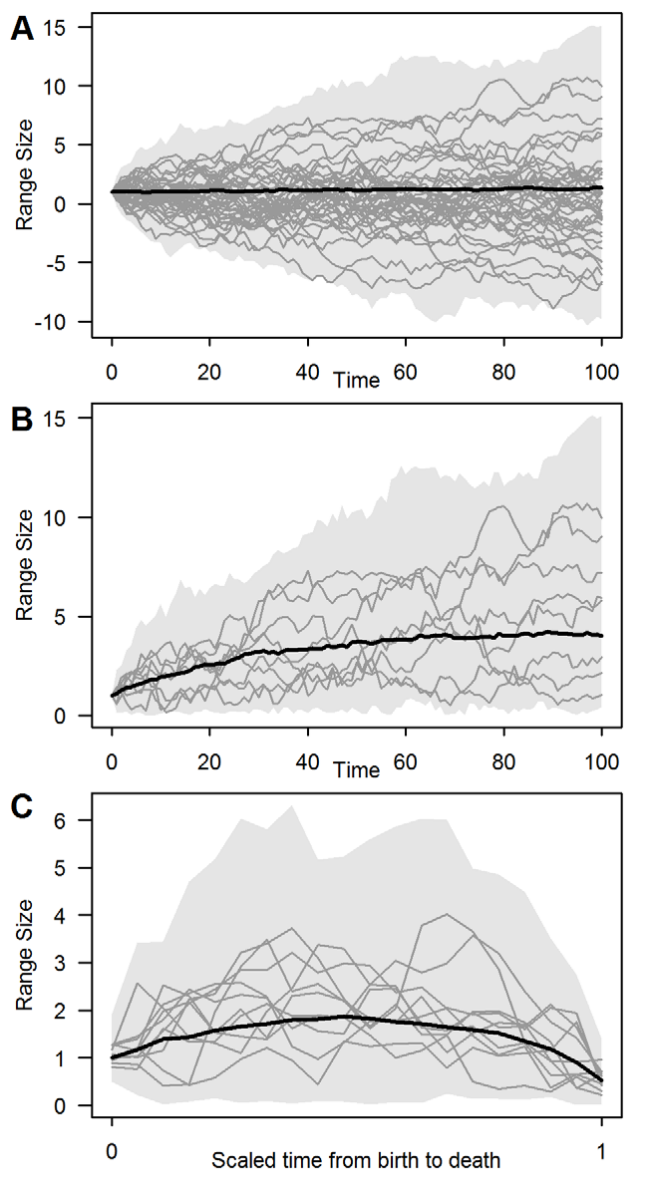
The role of censoring bias in generating the apparent trends in range size evolution. Range size trajectories (arbitrary units) are shown for (A) 1,000 species undergoing a random walk (μ = 0 and σ^2^ = 0.16) for 100 time steps, and for those species that either (B) survived to the present or (C) went extinct before the end of the simulation. To highlight the underlying random nature of this process, in (A) we continue to model the range trajectories of species even after they have gone extinct. Grey shading shows the extreme range size values through time, with the black line showing the median trend. The trajectories of a random selection of all species (grey lines), and of these, those surviving to the present are highlighted in (A) and (B), respectively. In (C) only those extinct lineages with a duration exceeding 20 time steps are included, with their time from birth to death scaled to between 0 and 1.

Directional trends between range size and species age in both molecular phylogenies and the fossil record have generally been interpreted as evidence that species undergo a predictable sequence of geographic expansion and contraction over the course of their life [7]–[14],[37]. However, using a stochastic model of range evolution we show that trends in range size with evolutionary age are expected even if range sizes have evolved randomly through time. Across the tips of reconstructed phylogenies, significant age–area correlations arise because of both the process of geographic speciation and the censoring of small-ranged species that went extinct before the present (Figure 3A and 3B). For extinct species, range sizes may appear to vary predictably with evolutionary age because of the censoring of those species that either survived to the present or were too rare to be detected (Figure 3). Our results demonstrate that correlations between range size and species age cannot be reliably interpreted as evidence for deterministic models of range evolution that predict directional trends in range size through time [1]–[4],[17].

Previous studies have interpreted a positive relationship between range size and evolutionary age as evidence that geographic ranges tend to expand through time [11],[13], apparently vindicating Willis's [1] theory of “age and area”. Here we show that this same pattern is also expected under a stochastic model because, when range size undergoes a random walk through time, only those species that, by chance, happened to expand their ranges or were initially widespread are likely to have survived to the present (Figures 1A, 1B, 3A, and 3B). Given the evidence from the fossil record for high rates of extinction [41] and the dependence of these rates on range size [42],[43], we suggest that extinction may be a more parsimonious explanation than any underlying trend for range expansion per se.

The illusion that geographic ranges tend to expand through time can also arise from the division of geographic ranges during speciation (Figure 1B). In this case the patterns are particularly misleading, for while average range sizes within the clade may be declining across each speciation event [27], an age–area correlation would instead suggest that ranges have been expanding through time.

Negative relationships between range size and evolutionary age have commonly been interpreted as evidence for a taxon cycle model [17], in which ranges rapidly expand following speciation before gradually contracting to extinction [11],[12],[14]. Our results show that a negative age–area correlation can also arise in the absence of any intraspecific range size trends, when newly formed species are geographically restricted but have a low probability of either speciating or going extinct (Figure 1A). These conditions may, in fact, be most likely to occur on island archipelagos, where taxon cycles have been most commonly invoked (e.g., [4],[17],[44]). Here, a species from the mainland gives rise to a number of daughter species, each endemic to an individual or small number of islands, and these persist for long periods of time in isolation [45]. Our results show that a negative relationship between range size and evolutionary age need not imply taxon cycle dynamics.

We might expect the fossil record to provide a more reliable signal of range evolution than molecular phylogenies, which rely on inferring these dynamics from a comparison of present-day range sizes [46]. Our analysis suggests, however, that interpreting the patterns in the fossil record is equally challenging because it, too, is likely to be biased with respect to geographic range size. First, when newly formed species are geographically restricted, as expected under a variety of speciation models [47], then only those species that, by chance, happened to expand their distributions are likely to be sampled. In contrast, those species that immediately declined to extinction are unlikely to be detected [28],[29] or will be represented in only one or a few sampling intervals (“singletons”), precluding an analysis of their range dynamics [7],[8]. As in molecular phylogenies, this leads to the illusion that geographic ranges have a tendency to expand post-speciation (Figure 3). Second, studies examining the fossil record have often excluded extant species on the basis that their full histories have yet to be played out [7],[8]. By excluding those species that survived to the present, a subsequent decline of geographic range size becomes inevitable (Figure 3). Our results suggest that, together, these two sampling biases can generate the pattern of “rise and fall” so frequently reported in the fossil record (Figures 2C, 2D, and 3) [7]–[10],[20].

One of the most surprising aspects of our simulations is the wide variety of relationships between range size and evolutionary age that can be generated by varying only a few biological parameters. For instance, when rates of range size evolution are slow, then—depending on the asymmetry of range division during speciation—our model predicts both negative and positive age–area correlations across extant species (Figure 2A) and negative and hump-shaped relationships amongst extinct species (Figure 2C). Differences in rates of range evolution or in the geography of speciation may therefore explain the heterogeneity in empirical age–area relationships reported both in previous and the current analyses [11]–[15],[24]. Our results also show, however, that even if all clades had evolved under the same conditions, substantial heterogeneity in age–area relationships can arise simply due to chance (Figure 2). Under some regions of parameter space this stochasticity is able to capture much of the variation observed across the different taxonomic groups (e.g., high rates of range size evolution, asymmetric range division, and when probabilities of speciation are independent of range size), whereas in other regions the match is extremely poor. For instance, patterns resembling those observed across extinct marine mollusks can be produced by a stochastic model only when range inheritance is highly asymmetric, as expected under a peripatric or micro-vicariance model of speciation. Independently derived estimates of these key parameters will therefore be required to assess the extent to which a single global model, versus a model with varying conditions across clades, is able explain the observed patterns.

Here we have focused on the range dynamics of individual species, but it has also been suggested that the areas occupied by entire clades may undergo regular patterns of expansion and contraction through time (e.g., [48]–[51]). Our results raise the possibility that these higher level dynamics may also not require deterministic explanations. Testing whether the geographic distributions of entire clades depart from a null expectation will require a more complex, spatial version of the model employed here that can account for both species' range sizes and their geographic overlap [27].

That patterns resembling the empirical data can arise from a random model does not imply that changes in species distributions occur at random [52]. Rather, the processes regulating geographic range size may be so complex and subject to historical contingency that the patterns of range evolution across species appear random [6]. Alternatively, even if species ranges did vary predictably through time the diagnostic tests that we have used may not be sufficiently refined to detect this. One reason for this may be that definitions of species age do not correspond to a realistic estimate of the evolutionary time from the birth to the death of a lineage [53]. For instance, under a peripatric model of speciation, the age of the parent species in a reconstructed phylogeny is reset to zero every time a dispersal event gives rise to a new species. Furthermore, given the sensitivity of the stochastic model to the underlying biological assumptions, stronger tests of departure from the null expectation will require constraining the possible parameter space based on a priori knowledge of the particular focal group. Regardless of these assumptions, however, our findings demonstrate that under a biologically realistic null model of range evolution, range sizes should not be expected to be independent of species age.

## Materials and Methods

### Stochastic Model of Range Size Evolution

We used a discrete time simulation of species diversification and range size evolution, starting from a single species with a range size drawn from an empirically realistic [54] log-normal distribution (log mean μ = 1, log variance σ^2^ = 1, 95% CI = [0.4, 19.3]). At each time step of the simulation, changes in range size for each extant species were drawn from a normal distribution with μ = 0, with extinction occurring if the range size of a species drifted to or below zero. By modifying the standard deviation of the distribution, we modelled the effects of differences in the rate of range size evolution (σ^2^ = 0.0001, 0.01, 0.04, 0.09, and 0.16), and the extremes of these rates lead, respectively, to extinction of 0% and >90% of species by the end of the simulation.

At each time step of the simulation, speciation could occur with a per-lineage probability ν. We simulated two speciation models, both of which have strictly bifurcating speciation. In the first model, ν varied as a set proportion of range size (0.005), corresponding to a value of ν = 0.5 when range size equalled 100 units. Such an increase in the probability of speciation with range size is expected under certain probabilistic models of range splitting when range sizes are strongly right skewed [27],[55],[56]. To prevent runaway clade growth associated with large range sizes, we truncated the range size used to calculate ν at 100. However, achieving such range size is rare over the lifetime of the simulation, even under the greatest degree of range change. In the second model, ν was equal across lineages and constant through time (ν = 0.02), analogous to the birth–death model [57],[58]. This latter value of ν was chosen to result in similar rates of speciation as expected in the range-size-dependent speciation model assuming the initial log-normal range size distribution. Varying the value of ν (0.014, 0.02, and 0.033) in the range-size-independent model or the rate at which ν increased with range size (0.0025, 0.005, and 0.01) did not qualitatively alter our results (Figures S2 and S3).

Upon speciation the geographic range of the parent was split amongst the two daughter lineages, with relative range sizes as a proportion (p) of the parent (p and 1−p) drawn from a beta distribution (α = 1 and β = 1, 5, 10, and 70). Increasing values of β resulted in more asymmetric splits, allowing us to model a variety of geographic scenarios from vicariance (β = 1) to peripatry and micro-vicariance (β = 70, 95% CI of p = [0.0003, 0.05]) [31],[32],[38],[59].

For each combination of parameter values we generated 500–5,000 replicate clades contingent on either the survival of at least six species to the present (the minimum richness in our empirical dataset of phylogenetic trees; Table S2) or the extinction of at least one species (the minimum required for comparison to our empirical fossil dataset). The number of replicates varied across parameter space because of the variation in computational time arising from differences in expected clade size and the probability of obtaining an extinct species. In total we simulated over 1 million clades. For each clade we obtained the reconstructed phylogeny of those species extant at the end of the simulation and recorded the age and area of extant species. In a reconstructed phylogeny, the age of a species is defined as the tip length: the time from the present to the most recent node. This estimate of species age is thus sensitive to the extinction of other lineages and does not account for the possibility of ancestral persistence during speciation. However, these limitations are common to both the observed and simulated phylogenies and so will not bias our results.

### Avian and Mammalian Phylogenies

We constructed trees based on mitochondrial protein coding genes (*cytb*, *COI*, *COII*, *ND2*) for genera and families of birds and mammals according to standard avian [60] and mammalian taxonomies [61]. Our selection of bird phylogenies largely follows that of Phillimore and Price [36], but we included additional sequences and species and an expanded taxonomic coverage where possible. Sequences were downloaded from GenBank and aligned by eye in MEGA v4 [62]. Trees were constructed using a relaxed clock Bayesian method in BEAST v1.5.4 [63]. For each tree we specified a Yule prior on branching times and that variation in rates of substitution amongst branches was uncorrelated and followed a log-normal distribution. For mammals we used an HKY model of substitution with four gamma categories and separate rates for codon positions 1+2 versus 3. Rates of molecular evolution are known to vary substantially amongst mammalian lineages [64], and so for each clade, trees were dated using fossil calibrations obtained from the literature. On all calibrations we used a log-normal prior on the age of the split with a mean and standard deviation of one and with the minimum age set to that used in the original study; usually corresponding to the first appearance of a group [65]. For birds we used a GTR-γ model of substitution, assuming an average rate of sequence substitution of 0.01 per site per lineage per million years [66]. We performed two runs of 5–10 million generations depending on the time required for convergence as assessed in Tracer v1.4 [67]. The first 10% of the generations were discarded as burn-in, and then, depending on the length of the model run, we sampled every 4,000–8,000 generations to produce a posterior distribution of trees. The trees were combined from the separate runs and summarized as the maximum clade credibility tree, from which we estimated species age as the tip length corresponding to each extant species. In three avian genera (*Cinclodes*, *Hemispingus*, and *Muscisaxicola*), runs failed to converge, and so in these cases we used an HKY model of substitution. Although this simpler model will tend to overestimate species ages, the frequency of significant age–area correlations across clades was similar when we re-ran all trees using the HKY model, showing that our results are robust to model assumptions. All trees are provided in Dataset S1.

Estimates of species ages from clades with large numbers of missing extant species will be relatively inflated, and so from our trees we included only those genera containing at least six species and where at least 80% of the species had been sampled. These clades generally corresponded to monophyletic, taxonomically recognized genera [60],[61], but we also included polyphyletic clades if at least 80% of the species from each constituent genera had been sampled. In total, we obtained estimates of species ages for 1,269 species from 64 genera across 17 orders (Table S1). For each species, we obtained the range size (km^2^) from previously published range databases of birds [68] and mammals [34].

### Age–Area Correlations

Because range sizes and species ages tend to be positively skewed within clades, we log-transformed both variables prior to analysis. For each clade we fit a quadratic model simplifying to a linear and then null model on the basis of an F-test. We aggregated the results of these models into five broad classes (Figure S1). Linear models provide three classes: no relationship (Type 1) and increasing (Type 2) and decreasing (Type 3) range size with species age. Within the range of the clade age data, some quadratic models show marked intermediate peaks (Type 4) or troughs (Type 5). However, where the vertex of the quadratic model is not central within the clade age data, quadratic models may describe predominantly increasing (Type 2) and decreasing (Type 3) range size with species age. We therefore assigned quadratic models to model Types 2–5 based on the location of the model vertex within terciles of the clade age data (Figure S1; Table 1). Our results are qualitatively similar if these classes of quadratic model are kept separate from linear models (Figures S1 and S4; Table S1).

### Fossil Mollusks

Data on the occupancy trajectories of extinct Cenozoic marine mollusks of New Zealand were obtained from Table S2 in Foote et al. [7]. The underlying data (from the Fossil Record Electronic Database; http://www.fred.org.nz) contains species records for many collection sites dated to geological stages: species range size was estimated as the proportion of collections within each geological stage that contained a species. To reduce sampling biases and to restrict analyses to extinct species, Foote et al. [7] restricted their dataset to species recorded from at least three contiguous stages and excluded all species with a Holocene record. In total, 140 species were used in the Foote et al. [7] analysis, and we refer the reader to their paper for further details on the methods.

For each species we split its duration into thirds and calculated the mean occupancy across the stages occurring within each of these three time bins. Because occupancy is given in discrete stages, these cannot always be divided evenly amongst the larger time bins (e.g., when the species is present in four, five, or seven time stages). In these cases we sought the most equitable distribution of bin lengths and randomly assigned these to each bin. For instance, a species with a duration of four time stages could have bin lengths of (1,1,2), (1,2,1), or (2,1,1). We repeated this procedure 1,000 times and calculated the mean percentage of species whose occupancy peaked in each time bin.

## Supporting Information

Dataset S1 **Bird and mammal phylogenetic trees.**
(ZIP)

Figure S1 **Classification of curve shapes from the regression of log range size on log species age.** Of the quadratic models, curve shapes are classified using the sign of the quadratic coefficient and the position of the curve vertex in relation to the observed species age values (grey panels). The corresponding models under the nine-category classification scheme used in Table S1 and Figure S4 are shown in brackets.
(TIFF)

Figure S2 **The relationship between the range size and the age of extant species expected under the stochastic model when rates of speciation (ν) are low.** Variation in Spearman's correlation (ρ) between species' age and geographic range area under different combinations of asymmetry in range size inheritance and rate of change in range size where probability of speciation (ν) increases with range size (ν = range size×0.0025) (A) or is constant (ν = 0.014) (B). Correlations are across all simulated clades for a particular parameter combination. Using a subsample of clades within an example combination (marked with an asterisk) shows that the observed correlation is strongly dependent on sample size (C). Grey boxes highlight the area of parameter space presented in Figure 2.
(TIFF)

Figure S3 **The relationship between the range size and the age of extant species expected under the stochastic model when rates of speciation (ν) are high.** Variation in Spearman's correlation (ρ) between species' age and geographic range area under different combinations of asymmetry in range size inheritance and rate of change in range size where probability of speciation (ν) increases with range size (ν = range size×0.01) (A) or is constant (ν = 0.033) (B). Correlations are across all simulated clades for a particular parameter combination. Using a subsample of clades within an example combination (marked with an asterisk) shows that the observed correlation is strongly dependent on sample size (C). Grey boxes highlight the area of parameter space presented in Figure 2.
(TIFF)

Figure S4 **The relative proportions of observed and expected age–area trajectories across reconstructed phylogenies and extinct species using nine model types.** The grey bars show the 95% confidence limits of the expected relative proportion of different age–area relationship classes under different combinations of asymmetry and range size inheritance for extant vertebrates (A and B) and extinct molluscs (C and D). The probability of speciation (ν) increases with range size (A and C) or is constant (B and D). Observed proportions of each class are shown as black bars for vertebrate orders (Table S1) and mollusk species (Table S3); shorter bars in (A) and (B) show the proportions for bird (left) and mammal (right) orders separately. The nine panels in each block correspond to the highlighted areas of Figure 1.
(TIFF)

Table S1 **Age–area relationships across clades of birds and mammals with relationships classified into nine types.**
(DOC)

Table S2 **Age–area relationships for individual clades of birds and mammals.**
(DOC)

Table S3 **Range trajectories for fossil mollusks.**
(DOC)
