# Supplementary figures and images for: Speciation and Extinction Drive the Appearance of Directional Range Size Evolution in Phylogenies and the Fossil Record
Source: PLoS Biol. 2012 Feb 21;10(2):e1001260. doi: 10.1371/journal.pbio.1001260 (PMC3283545; doi:10.1371/journal.pbio.1001260)

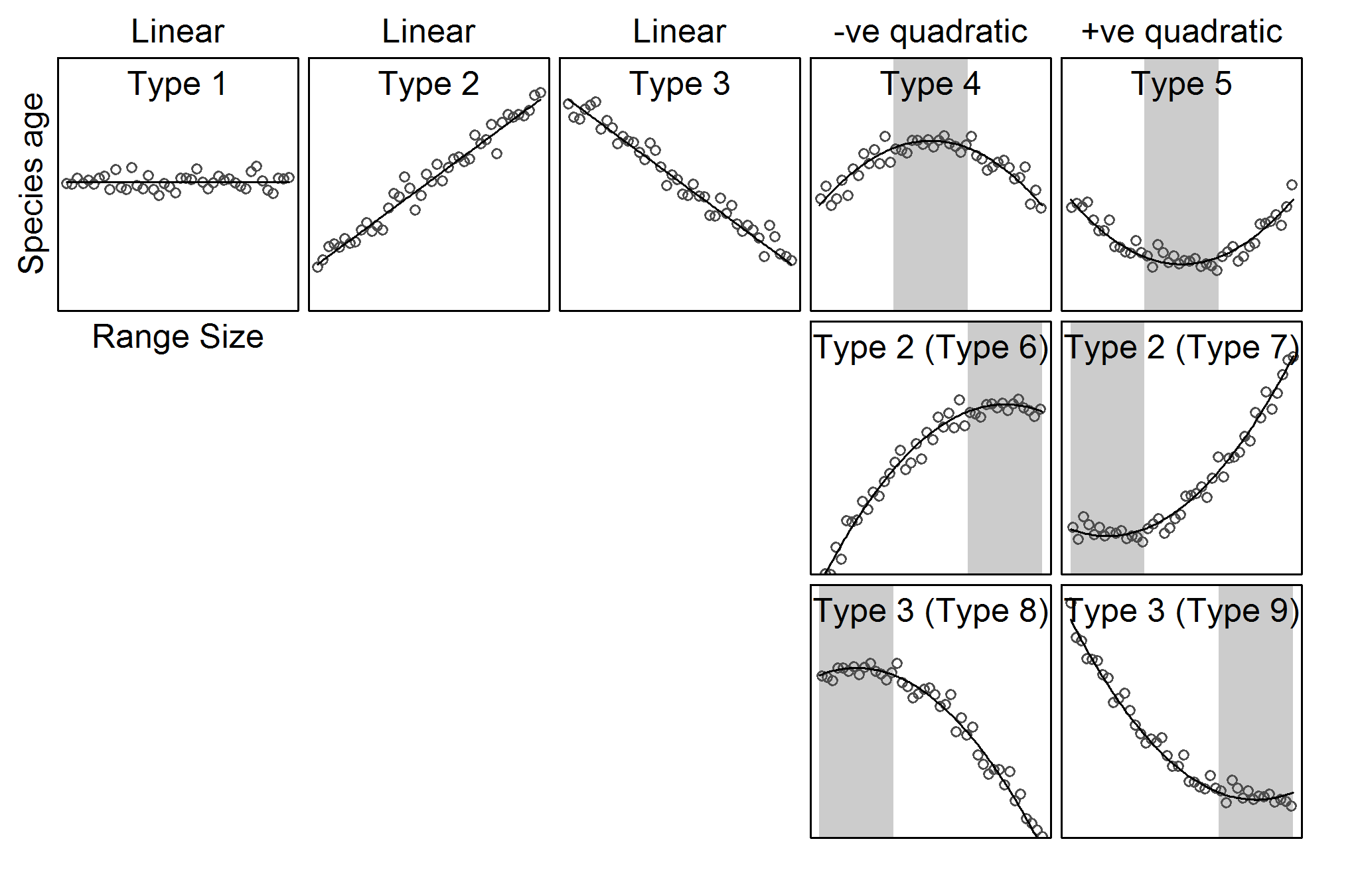

Supplement: Figure S1 — Classification of curve shapes from the regression of log range size on log species age. Of the quadratic models, curve shapes are classified using the sign of the quadratic coefficient and the position of the curve vertex in relation to the observed species age values (grey panels). The corresponding models under the nine-category classification scheme used in Table S1 and Figure S4 are shown in brackets. (TIFF) [file pbio.1001260.s002.tiff]

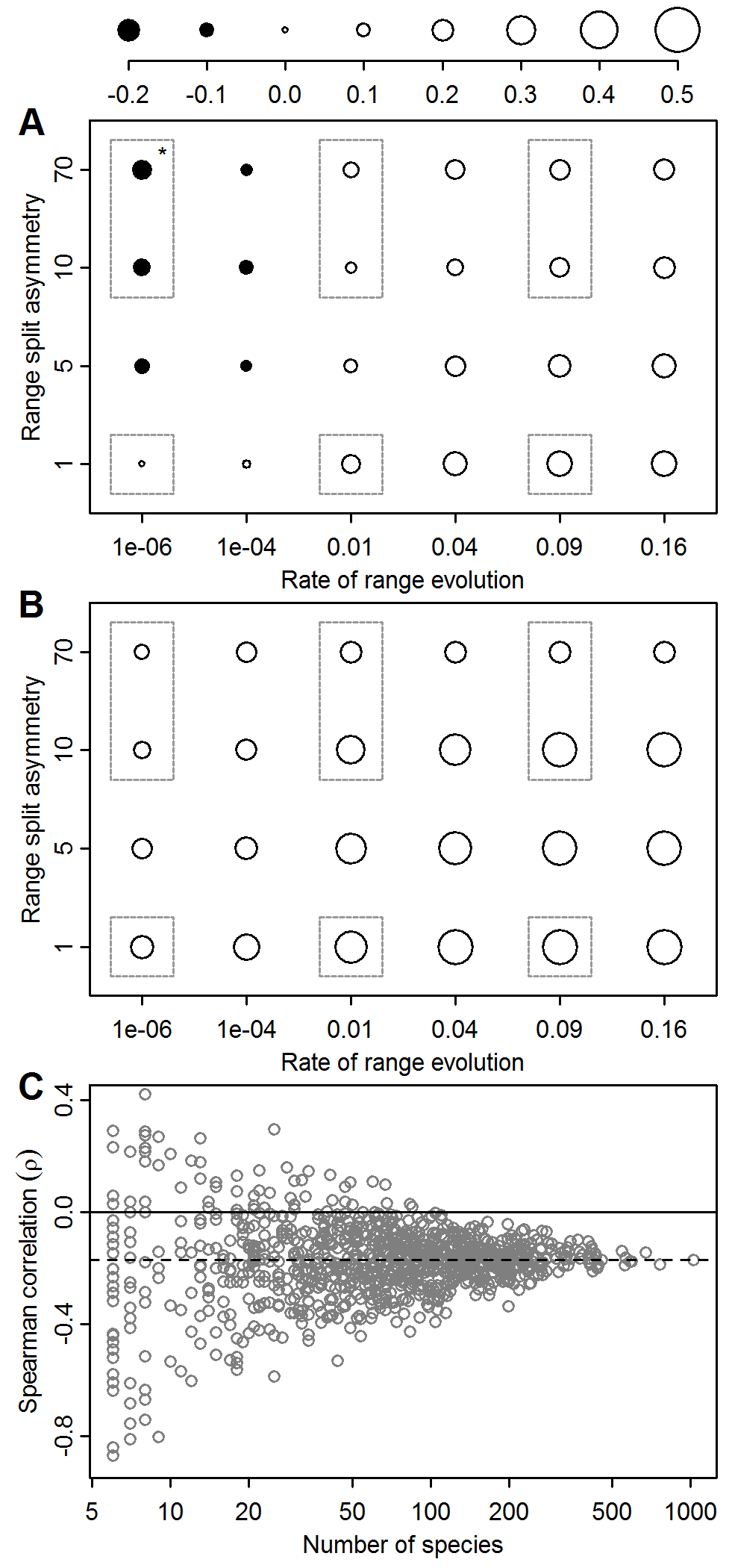

Supplement: Figure S2 — The relationship between the range size and the age of extant species expected under the stochastic model when rates of speciation (ν) are low. Variation in Spearman's correlation (ρ) between species' age and geographic range area under different combinations of asymmetry in range size inheritance and rate of change in range size where probability of speciation (ν) increases with range size (ν = range size×0.0025) (A) or is constant (ν = 0.014) (B). Correlations are across all simulated clades for a particular parameter combination. Using a subsample of clades within an example combination (marked with an asterisk) shows that the observed correlation is strongly dependent on sample size (C). Grey boxes highlight the area of parameter space presented in Figure 2. (TIFF) [file pbio.1001260.s003.tiff]

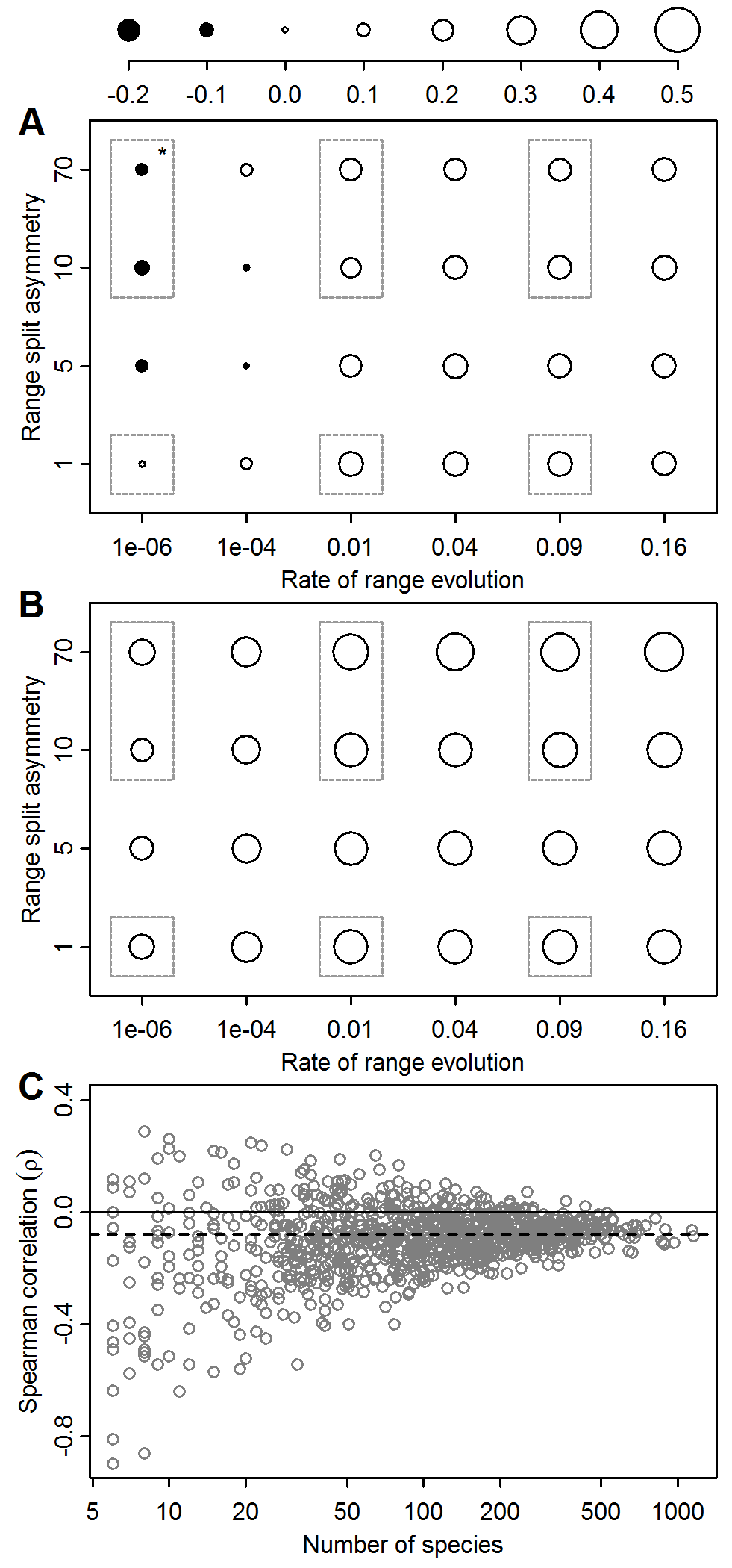

Supplement: Figure S3 — The relationship between the range size and the age of extant species expected under the stochastic model when rates of speciation (ν) are high. Variation in Spearman's correlation (ρ) between species' age and geographic range area under different combinations of asymmetry in range size inheritance and rate of change in range size where probability of speciation (ν) increases with range size (ν = range size×0.01) (A) or is constant (ν = 0.033) (B). Correlations are across all simulated clades for a particular parameter combination. Using a subsample of clades within an example combination (marked with an asterisk) shows that the observed correlation is strongly dependent on sample size (C). Grey boxes highlight the area of parameter space presented in Figure 2. (TIFF) [file pbio.1001260.s004.tiff]

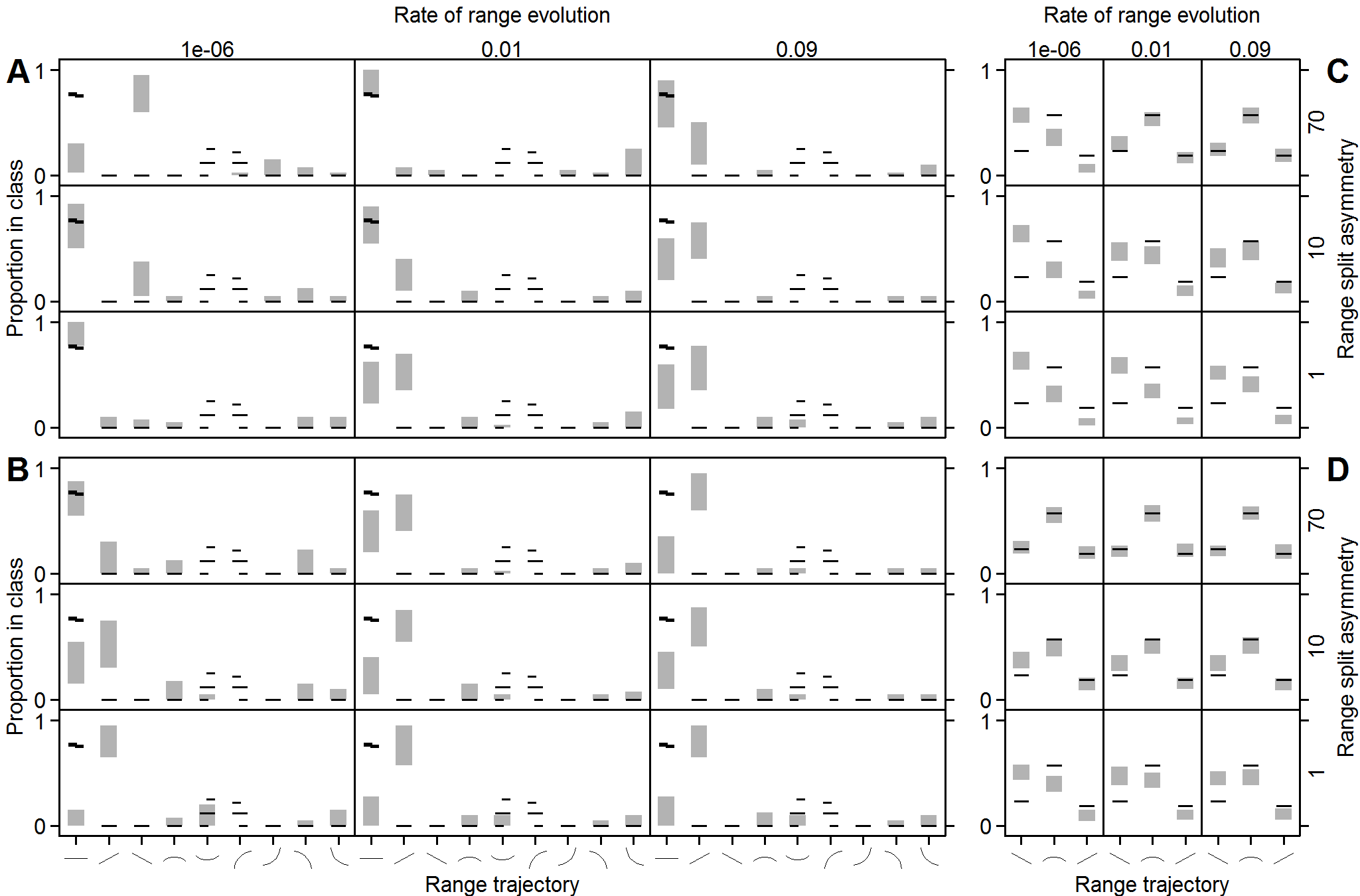

Supplement: Figure S4 — The relative proportions of observed and expected age–area trajectories across reconstructed phylogenies and extinct species using nine model types. The grey bars show the 95% confidence limits of the expected relative proportion of different age–area relationship classes under different combinations of asymmetry and range size inheritance for extant vertebrates (A and B) and extinct molluscs (C and D). The probability of speciation (ν) increases with range size (A and C) or is constant (B and D). Observed proportions of each class are shown as black bars for vertebrate orders (Table S1) and mollusk species (Table S3); shorter bars in (A) and (B) show the proportions for bird (left) and mammal (right) orders separately. The nine panels in each block correspond to the highlighted areas of Figure 1. (TIFF) [file pbio.1001260.s005.tiff]
