# Supplementary material for: Speciation and Extinction Drive the Appearance of Directional Range Size Evolution in Phylogenies and the Fossil Record
Source: PLoS Biol. 2012 Feb 21;10(2):e1001260. doi: 10.1371/journal.pbio.1001260 (PMC3283545; doi:10.1371/journal.pbio.1001260)
Supplement: Table S1 — Age–area relationships across clades of birds and mammals with relationships classified into nine types. (DOC) [file pbio.1001260.s006.doc]

**Table S1 Age-area relationships across clades of birds and mammals with relationships classified into 9 types.** The percentage of total taxa falling into each relationship class is given along with the percentages of mammal and bird taxa in brackets. Results are presented for individual genera and for genera combined into orders, along with the number of clades (N) and their median species richness (S). See Figure S1 for classification of age-area relationships.

| Age-area class | Genera | | Orders | |
| --- | --- | --- | --- | --- |
|  | 90.6 | (93.3,88.2) | 76.5 | (75, 77.8) |
|  | 3.1 | (3.3,2.9) | 0 | (0,0) |
|  | 0 | (0,0) | 0 | (0,0) |
|  | 0 | (0,0) | 0 | (0,0) |
|  | 4.7 | (0,8.8) | 11.8 | (25,0) |
|  | 0 | (0,0) | 11.8 | (0,22.2) |
|  | 1.6 | (3.3,0) | 0 | (0,0) |
|  | 0 | (0,0) | 0 | (0,0) |
|  | 0 | (0,0) | 0 | (0,0) |
| N | 64 | (30,34) | 17 | (8,9) |
| S | 12 | (9.5,12) | 29 | (59,24) |
