# Supplementary material for: Speciation and Extinction Drive the Appearance of Directional Range Size Evolution in Phylogenies and the Fossil Record
Source: PLoS Biol. 2012 Feb 21;10(2):e1001260. doi: 10.1371/journal.pbio.1001260 (PMC3283545; doi:10.1371/journal.pbio.1001260)
Supplement: Table S2 — Age–area relationships for individual clades of birds and mammals. (DOC) [file pbio.1001260.s007.doc]

**Table S2. Age-area relationships for individual clades of birds and mammals.**

| Order | Genus | Species richness* | Age-area class |
| --- | --- | --- | --- |
|
| *Anseriformes* | *Anas* | 39 | 1 |
| *Anseriformes* | *Anser* | 10 | 1 |
| *Anseriformes* | *Aythya* | 9 | 1 |
| *Anseriformes* | *Cygnus* | 6 | 1 |
| *Anseriformes* | *Oxyura* | 7 | 1 |
| *Anseriformes* | *Tadorna* | 6 | 1 |
|  |  | 116 | 2 |
| *Ciconiiformes* | *Tringa* | 15 | 1 |
| *Craciformes* | *Crax* | 7 | 1 |
| *Craciformes* | *Mitu* | 7 | 1 |
|  |  | 23 | 1 |
| *Gruiformes* | *Grus* | 13 | 1 |
|  |  | 15 | 1 |
| *Musophagiformes* | *Tauraco* | 22 | 1 |
| *Passeriformes* | *Acanthiza* | 12 | 1 |
| *Passeriformes* | *Amytornis* | 8 | 1 |
| *Passeriformes* | *Catharus* | 12 | 1 |
| *Passeriformes* | *Cinclodes* | 12 | 1 |
| *Passeriformes* | *Dendroica* | 30 | 1 |
| *Passeriformes* | *Empidonax* | 15 | 1 |
| *Passeriformes* | *Ficedula* | 25 | 1 |
| *Passeriformes* | *Geositta* | 11 | 1 |
| *Passeriformes* | *Geothlypis* | 13 | 1 |
| *Passeriformes* | *Hemispingus* | 10 | 1 |
| *Passeriformes* | *Hirundo* | 35 | 1 |
| *Passeriformes* | *Muscisaxicola* | 12 | 5 |
| *Passeriformes* | *Myioborus* | 12 | 5 |
| *Passeriformes* | *Progne* | 6 | 1 |
| *Passeriformes* | *Tachycineta* | 8 | 1 |
| *Passeriformes* | *Tangara* | 45 | 1 |
| *Passeriformes* | *Thamnophilus* | 25 | 1 |
| *Passeriformes* | *Turdus* | 67 | 1 |
| *Passeriformes* | *Vermivora* | 11 | 1 |
|  |  | 422 | 2 |
| *Piciformes* | *Pteroglossus* | 12 | 2 |
| *Piciformes* | *Ramphastos* | 11 | 5 |
|  |  | 24 | 1 |
| *Psittaciformes* | *Amazona* | 29 | 1 |
| *Trogoniformes* | *Trogon* | 17 | 1 |
|  |  | 28 | 1 |
| *Artiodactyla* | *Bos* | 6 | 1 |
| *Artiodactyla* | *Capra* | 8 | 1 |
| *Artiodactyla* | *Cephalophus* | 14 | 1 |
| *Artiodactyla* | *Gazella* | 7 | 1 |
| *Artiodactyla* | *Martes* | 7 | 1 |
| *Artiodactyla* | *Mustela* | 15 | 1 |
| *Artiodactyla* | *Tragelaphus* | 9 | 1 |
|  |  | 83 | 1 |
| *Carnivora* | *Genetta* | 13 | 1 |
|  |  | 57 | 5 |
| *Chiroptera* | *Artibeus* | 18 | 1 |
| *Chiroptera* | *Platyrrhinus* | 8 | 1 |
| *Chiroptera* | *Sturnira* | 11 | 1 |
| *Chiroptera* | *Vampyressa* | 6 | 1 |
|  |  | 62 | 1 |
| *Dasyuromorphia* | *Antechinus* | 8 | 1 |
| *Dasyuromorphia* | *Dasyurus* | 6 | 1 |
| *Dasyuromorphia* | *Pseudantechinus* | 6 | 1 |
| *Dasyuromorphia* | *Sminthopsis* | 18 | 1 |
|  |  | 61 | 1 |
| *Lagomorpha* | *Ochotona* | 27 | 1 |
| *Primates* | *Alouatta* | 9 | 1 |
| *Primates* | *Hylobates* | 7 | 1 |
| *Primates* | *Macaca* | 20 | 1 |
|  |  | 43 | 5 |
| *Rodentia* | *Calomys* | 10 | 1 |
| *Rodentia* | *Chaetodipus* | 16 | 1 |
| *Rodentia* | *Dipodomys* | 19 | 1 |
| *Rodentia* | *Eothenomys* | 7 | 1 |
| *Rodentia* | *Marmota* | 14 | 2 |
| *Rodentia* | *Microtus* | 51 | 1 |
| *Rodentia* | *Perognathus* | 9 | 1 |
| *Rodentia* | *Spermophilus* | 38 | 1 |
| *Rodentia* | *Tamias* | 25 | 2 |
|  |  | 218 | 1 |
| *Soricomorpha* | *Talpa* | 8 | 1 |
|  |  | 24 | 1 |

*The number of species within each order may exceed that of the labeled genera because in the order level analysis we also included age estimates from genera with less than 6 species.
