# Supplementary material for: Speciation and Extinction Drive the Appearance of Directional Range Size Evolution in Phylogenies and the Fossil Record
Source: PLoS Biol. 2012 Feb 21;10(2):e1001260. doi: 10.1371/journal.pbio.1001260 (PMC3283545; doi:10.1371/journal.pbio.1001260)
Supplement: Table S3 — Range trajectories for fossil mollusks. (DOC) [file pbio.1001260.s008.doc]

**Table S3 Range trajectories for fossil mollusks.** Results show the mean percentage of species whose average range size peaked in the first, second or third tercile of their duration.

| Tercile | % of species (95% confidence interval) |
| --- | --- |
| 1 | 22.8 (20.7, 25) |
| 2 | 57.4 (54.2, 60.7) |
| 3 | 19.0 (16.4, 22.1) |
